# Activation of branched chain amino acid catabolism protects against nephrotoxic acute kidney injury

**DOI:** 10.1152/ajprenal.00260.2024

**Published:** 2024-12-10

**Authors:** Samaneh DiMartino, Monica P. Revelo, Sandeep K. Mallipattu, Sian E. Piret

**Affiliations:** ^1^Division of Nephrology and Hypertension, Department of Medicine, Stony Brook University, Stony Brook, New York, United States; ^2^Department of Pathology, University of Utah, Salt Lake City, Utah, United States; ^3^Renal Section, Northport VA Medical Center, Northport, New York, United States

**Keywords:** acute kidney injury, branched chain amino acids, cellular metabolism, proximal tubule

## Abstract

Acute kidney injury (AKI) is a major risk factor for chronic kidney disease (CKD), and there are currently no therapies for AKI. Proximal tubules (PTs) are particularly susceptible to AKI, due to nephrotoxins such as aristolochic acid I (AAI). Normal PTs use fatty acid oxidation and branched chain amino acid (BCAA; valine, leucine, and isoleucine) catabolism to generate ATP; however, in AKI, these pathways are downregulated. Our aim was to investigate the utility of a pharmacological activator of BCAA catabolism, BT2, in preventing nephrotoxic AKI. Mice were administered two injections of AAI 3 days apart to induce AKI, with or without daily BT2 treatment. Mice treated with BT2 had significantly protected kidney function (reduced serum creatinine and urea nitrogen), reduced histological injury, preservation of PT (Lotus lectin staining), and less PT injury (cytokeratin-20 staining) and inflammatory gene expression compared with mice with AAI alone. Mice with AKI had increased circulating BCAA and accumulation of BCAA in the kidney cortex. Leucine is a potent activator of the mechanistic target of rapamycin complex 1 (mTORC1) signaling, and mTORC1 signaling was activated in mice treated with AAI. However, BT2 reduced kidney cortical BCAA accumulation and attenuated the mTORC1 signaling. In vitro, injured primary PT cells had compromised mitochondrial bioenergetics, but cells treated with AAI + BT2 had partially restored mitochondrial bioenergetics and improved injury markers compared with cells treated with AAI alone. Thus, pharmacological activation of BCAA catabolism using BT2 attenuated nephrotoxic AKI in mice.

**NEW & NOTEWORTHY** This study explored the effects of pharmacological activation of branched chain amino acid (BCAA) catabolism using BT2 to prevent nephrotoxic acute kidney injury (AKI) in mice. Our results indicate that activation of BCAA catabolism protects against nephrotoxic AKI, in association with reduced BCAA accumulation, reduced mammalian target of rapamycin protein complex 1 signaling, and improved mitochondrial bioenergetics.

## Graphical abstract

**Figure:**
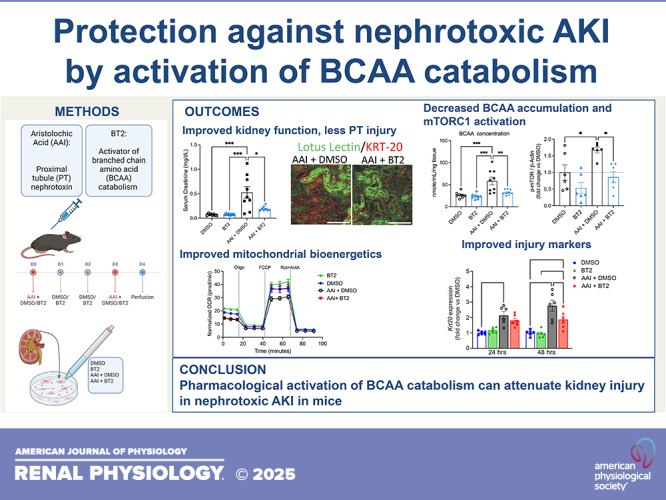

## INTRODUCTION

Acute kidney injury (AKI) causes significant morbidity and mortality, both in itself, and as a major risk factor for developing fibrosis and chronic kidney disease (CKD) (1). The proximal tubule (PT) is particularly susceptible to AKI, which may be caused by various factors, including ischemia-reperfusion, sepsis, and nephrotoxic DNA damaging agents such as environmental toxins [e.g., aristolochic acid I (AAI)] or chemotherapeutics (e.g., cisplatin) (2). During AKI, PTs undergo substantial changes in morphology and cellular biology, including dedifferentiation, cell cycle reentry, and alterations in metabolism (2). Following injury, PTs may repair or undergo arrest in the G2/M stage of the cell cycle, resulting in an ineffective or maladaptive repair state characterized by evidence of senescence, which is associated with the development of interstitial fibrosis and a subsequent decline in functional capabilities (3). PT cellular metabolism is affected by both direct mitochondrial damage and transcriptional downregulation of key metabolic enzymes (4). PTs predominantly use mitochondrial fatty acid β-oxidation (FAO) to generate ATP. During AKI, FAO is severely suppressed, likely contributing to injury (4, 5). Recent studies have shown that uninjured kidneys also catabolize branched chain amino acids (BCAAs: valine, leucine, and isoleucine) to generate tricarboxylic acid (TCA) cycle intermediates, likely predominantly in the PT (6, 7). However, we and others have shown that BCAA catabolism is also downregulated in nephrotoxic (AAI-induced), septic, and ischemia-reperfusion-induced AKI, leading to the accumulation of BCAA in the cortex (8–10).

BCAAs are used in protein synthesis, catabolism to acetyl-CoA and succinyl-CoA, and neurotransmitter synthesis (11). BCAAs are catabolized through a series of mitochondrial enzymatic reactions. After freely reversible transamination of BCAAs to branched chain α-keto acids (BCKAs) by branched chain aminotransferase (BCAT), BCKAs undergo irreversible decarboxylation catalyzed by the branched chain ketoacid dehydrogenase (BCKDH) complex, which also serves as the rate-limiting enzyme in this metabolic pathway (11). A further series of enzymatic reactions results in the production of acetyl-CoA and succinyl-CoA, which enter the TCA cycle. The BCKDH complex comprises multiple subunits of E1α, E1β, E2, and E3 proteins, with E1α exhibiting the catalytic activity responsible for the decarboxylation reaction (11). Regulation of BCKDH activity is achieved through phosphorylation and dephosphorylation of the E1α subunit, mediated by BCKDH kinase (BCKDK) and BCKDH phosphatase (PP2Cm), respectively. Phosphorylation is inhibitory, whereas dephosphorylation is activating, and this modulates the flux of BCAAs through the pathway (11). Dysregulation of BCKDH activity has been implicated in various metabolic disorders, including maple syrup urine disease and insulin resistance (11). Recently, BT2 (3,6-dichlorobenzothiophene-2-carboxylic acid) has emerged as a potential therapeutic agent capable of modulating BCAA catabolism. BT2 is an allosteric inhibitor of BCKDK which reduces the inhibitory phosphorylation of BCKDH-E1α and enhances flux through the BCAA catabolic pathway, facilitating BCAA entry into the TCA cycle (12–15). Activation of BCAA catabolism by BT2 has been used in studies of liver carcinoma, heart failure, and metabolic dysfunction (12, 16, 17).

Genes encoding BCAA catabolic enzymes, including *Bckdha* (BCKDH-E1α subunit) and *Bckdhb* (BCKDH-E1β subunit) are downregulated in AKI, and our aim was therefore to investigate the use of BT2 to prevent AKI. We used AAI as a model of nephrotoxic AKI, as we had previously shown downregulation of BCAA catabolic enzymes in this model (9). AAI is a PT-specific DNA damaging agent, which also causes mitochondrial damage and transition to fibrosis in mice (9, 18, 19). We hypothesized that BT2 would increase BCAA catabolic flux in PT cells. Furthermore, since leucine is a potent activator of the mechanistic target of rapamycin complex 1 (mTORC1) signaling, we hypothesized that BT2 may reduce leucine accumulation and lead to reduced mTORC1 activation in AKI.

## MATERIALS AND METHODS

### Animals

**Figure 1.**
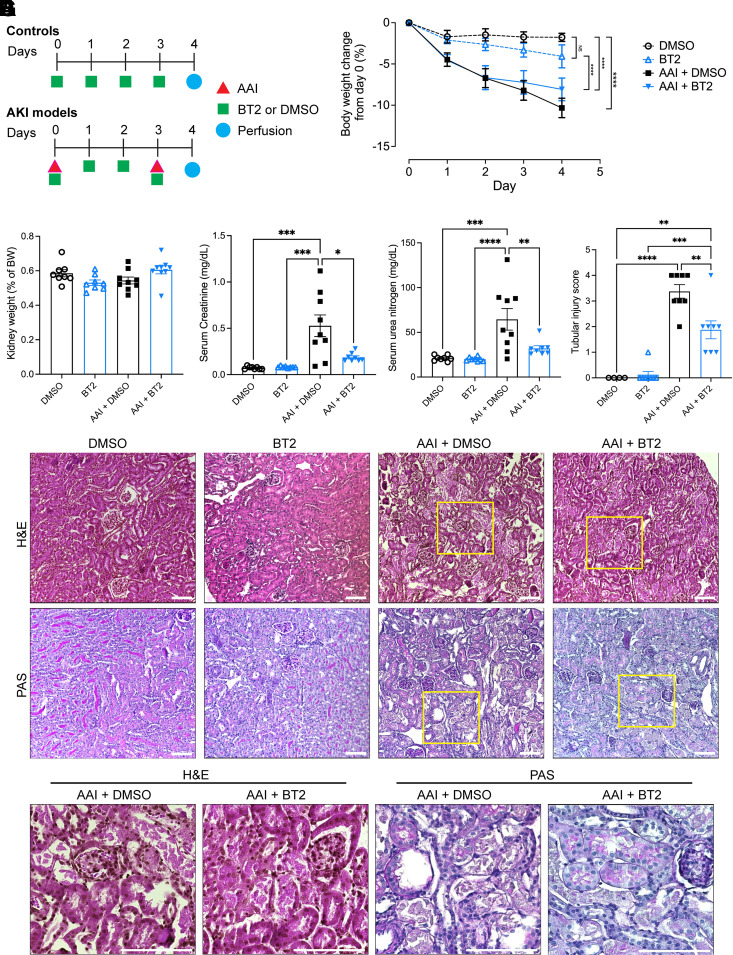
BT2 protects kidney function and histology after nephrotoxic AKI in mice. *A*: the experimental design for AAI/DMSO/BT2 treatments in different mice groups. *B*: percentage body weight changes from *day 0*. *n* = 8 or 9 per group. *C*: kidney weights as ratios to body weights. *D*: serum creatinine and (*E*) serum urea nitrogen concentrations. *F*: histological analysis of kidney cortex using hematoxylin and eosin (H&E) and periodic acid-Schiff (PAS) stains. Representative images provided from each group. Scale bars = 100 µm.* G*: tubular injury scoring. AKI, acute kidney injury; AAI, aristolochic acid I. *B*–*E, G*: *P < 0.05, **P < 0.01, ***P < 0.001, ****P < 0.0001, two-way ANOVA.

All animal studies were approved by Stony Brook University Institutional Animal Care and Use Committee. Mice were fed ad libitum and maintained at constant ambient temperature in a 12-h light-dark cycle. AKI was induced in 14–16-wk-old C57Bl/6 male mice by intraperitoneal injection of two doses of AAI at 3 mg/kg body weight on *days 0* and *3* of the experiment (Fig. 1*A*). DMSO (AAI + DMSO) or BT2 (Sigma-Aldrich) at 20 mg/kg per day (AAI + BT2) (13) were additionally injected daily. Control mice received daily injections of the same dose of DMSO or BT2. Body weight was recorded daily. Blood was collected 24 h after the last treatment under terminal anesthesia by cardiac puncture followed by cardiovascular perfusion with PBS. Kidneys were collected for analysis of RNA, protein, BCAA concentrations, and histology.

### Kidney Functional Analysis

Serum samples were submitted to the University of Alabama at Birmingham O’Brien Core Center to determine serum creatinine by isotope dilution LC-MS/MS. Serum urea nitrogen was measured using a colorimetric detection method (Arbor Assays) based on the manufacturer’s protocol.

### Histological Analysis

Kidneys were fixed in 10% phosphate-buffered formalin for 24 h, followed by processing, paraffin embedding, and sectioning at 4 µm thickness. Sections were stained with hematoxylin and eosin (H&E) or periodic acid-Schiff (PAS) (Sigma-Aldrich) based on standard protocols. Slides were visualized using a Nikon Eclipse 90i microscope and Nikon DS-Qi2 camera. Histologic scoring for tubular damage and inflammation was evaluated by a semiquantitative scale from 0 to 4 representing 0: no damage, 1: <25%, 2: 25%–50%, 3: 50%–75%, and 4: >75% by a histopathologist (M.P.R.) who was blinded to treatments. Features of tubular damage evaluated included: tubular dilatation, attenuation of tubular epithelial cells cytoplasm, intracytoplasmic vacuoles, and presence of luminal casts.

### RNA Extraction, cDNA Synthesis and Quantitative Reverse Transcriptase PCR

The kidney cortex was collected in RNAlater buffer (Sigma-Aldrich) and stored at −80°C. RNA from frozen tissue was extracted using the RNeasy kit (Qiagen), following the manufacturer’s protocol. For cells, total RNA was extracted using TRIzol (Gibco). Samples were treated with DNase, and reverse transcription was performed using a SuperScript VILO cDNA Synthesis kit according to instructions. Quantitative PCR was performed on the diluted cDNA in triplicates using Powerup SYBR Green qPCR master mix on an ABI QuantStudio 3 Real-Time PCR instrument (Applied Biosystems). Primers were designed using the Primer-BLAST tool from the National Center for Biotechnology Information and were validated for their efficiency before use (Supplemental Table S1). Expression levels were normalized to *Actb* and were calculated as fold change versus control groups using the comparative cycle threshold 2^−ΔΔCT^ method.

### Immunofluorescence Staining

Paraffin sections were deparaffinized and rehydrated, and antigen retrieval was performed using citrate buffer pH6. After blocking, sections were incubated with rabbit anti-cytokeratin-20 (ab97511; Abcam) at 4°C overnight. Then, sections were incubated with secondary goat anti-rabbit (111-005-144; Jackson ImmunoResearch) followed by tertiary fluorescently labeled donkey anti-goat-568 (A11057; Life Technologies). Subsequently, sections were stained with fluorescein-labeled Lotus lectin (Vector Laboratories) and Hoechst (Invitrogen). Coverslips were then mounted to slides with ProLong Gold Antifade mounting media. Digital images were obtained with a Nikon Eclipse 90i microscope and DS-Qi2 camera, and fluorescence was quantified as area-stained using ImageJ software (9).

### Western Blotting

Proteins from snap-frozen kidney cortex tissues were harvested in radioimmunopecipitation assay (RIPA) buffer [50 mM Tris-HCl pH 7.4, 150 mM NaCl, 10 mM NaF, 1 mM EDTA, 15 mM NP-40, 0.1% SDS, and 1× Halt protease and phosphatase inhibitor cocktail (Thermo Scientific)]. Protein concentrations were quantified using a Pierce BCA Protein Assay Kit (Thermo Scientific). The same amount of protein (20–50 µg) was separated by 10% Novex Tris-Glycine or 4%–12% gradient NuPAGE Bis-Tris precast gels (Thermo Scientific) and transferred to 0.22-µm nitrocellulose membranes (iBlot-3 Thermo Scientific). After blocking with 5% nonfat milk/Tris-buffered saline (TBS) or StartingBlock T20 (TBS) blocking buffer (Thermo Scientific) for 1 h at room temperature, membranes were incubated with primary antibodies (Supplemental Table S2) at 4°C overnight. Membranes were then incubated with appropriate secondary horseradish peroxidase (HRP)-conjugated antibodies at room temperature for 1 h followed by developing with chemiluminescence detection (Pierce ECL), Western blotting substrate (Thermo Scientific), or Immobilon Western Chemiluminescent HRP Substrate (Millipore). Relative intensity of the blots was quantified by ImageJ software, and results were normalized to β-Actin.

### Quantification of BCAA Concentrations in Serum and Kidney Cortex

BCAA concentrations in mouse serum and kidney cortex were measured through colorimetric assay using the Branched Chain Amino Acid Colorimetric Kit (MAK003; Sigma). In this assay, BCAA concentration is determined using a coupled enzyme reaction to catalyze the conversion of valine, leucine, and isoleucine to their respective ketoacids, which results in the conversion of NAD+ to NADH. NADH is detected by a colorimetric indicator, with an absorption maximum of 450 nm, proportional to the amount of BCAA present. Briefly, 10 mg kidney cortex tissue was weighed and homogenized in cold BCAA assay buffer. Samples were centrifuged, and supernatant was used for further analysis according to the manufacturer’s instructions. Leucine standards were used to generate standard curves. To remove the effect of NADH and NADPH background, a blank was set up for each sample and the blank readings were subtracted from the sample and standard readings. Results are expressed as nmol/mL (serum) or nmol/mL per mg tissue (cortex).

### Primary PT Cell Culture

Mouse primary PT cells were extracted from the kidney cortex of 3- to 5-wk-old C57Bl/6 male mice immediately after cardiovascular perfusion with PBS as previously described (19). The cortex was minced finely and then subjected to a 45-min incubation at 37°C in a solution containing 2 mg/mL of collagenase A, with gentle shaking at 750 rpm. Post digestion, the tissue was filtered through a 100-µm strainer twice and centrifuged, and the pellet was resuspended in PBS and a mixture of 5% fetal bovine serum/PBS, and finally transferred into PT cell media. The PT cell media comprised DMEM:F12 supplemented with 10 µg/L EGF, 5 pM T3, 3.5 mg/L ascorbic acid, 25 µg/L hydrocortisone, 1× insulin-transferrin-selenium supplement, 100 units/mL penicillin, and 100 µg/mL streptomycin. Cells were treated with either DMSO or AAI (25 µM), with or without BT2 (25 µM), and RNA harvested for qRT-PCR at 24 h and 48 h.

### Quantification of BCAA Concentrations in Primary PT Cells

BCAA concentrations in primary cell cultures were quantified using the BCAA-Glo Assay (Promega). This assay couples oxidation of leucine, isoleucine, and valine to their respective ketoacids plus NADH. In the presence of NADH, reductase enzymatically reduces a proluciferin reductase substrate to luciferin, which is detected using Ultra-Glo recombinant luciferase. The amount of light produced is proportional to the amount of BCAA in the sample. Briefly, cells were plated in 96-well white clear-bottom plates at 40,000 cells per well. After 24 h, cells were treated with either DMSO, 25 μM BT2, 25 μM AAI, or a combination of AAI and BT2. After a further 24 h, cells were washed three times with cold PBS and resuspended in 0.25 mL of PBS, followed by rapid lysis in 0.125 mL of 0.6 N HCl for 5 min and neutralization with 0.125 mL of neutralization buffer. The metabolites' detection reactions were started by the addition of 50 μL of BCAA detection reagent. Luminescence was read after a 1-h incubation at room temperature. In all experiments, a standard curve of two to four concentrations of leucine, including a high concentration of leucine (25 µM), a low concentration (1 µM) as well as a negative control (buffer only), was used. BCAA concentration in the sample was calculated using the following formula: (BCAA) = (Leucine standard, µM) × (RLU_sample_ − RLU_background_) (RLU_leucine standard_ − RLU_background_). To normalize BCAA concentrations, total protein concentration was measured using the Micro BCA Protein Assay Kit (Thermo Fisher Scientific) and BCAA concentrations reported as pmol/µg protein. BCAA concentrations were measured in three different sets of primary PT cells, each derived from separate mice.

### Assessment of Mitochondrial Bioenergetics in Primary PT Cells

Mitochondrial function was evaluated in live cells by measuring oxygen consumption rate (OCR) using an Agilent Seahorse XFe96 Analyzer and MitoStress Test with mitochondrial function inhibitors. Primary PT cells were seeded at a density of 20,000 cells per well. After 24 h, the growth medium was exchanged for serum-free Seahorse DMEM (pH 7.4) supplemented with 10 mM glucose, 1 mM glutamine, and 2 mM pyruvate. In addition, cells were treated with either DMSO, 25 μM BT2, 25 μM AAI, or a combination of AAI and BT2. After 24 h, the medium was renewed with fresh Seahorse DMEM of identical composition and MitoStress test was undertaken. The final concentrations of mitochondrial inhibitors used were 1.5 µM oligomycin, 3 µM FCCP, and 0.5 µM rotenone/antimycin A. OCR was normalized based on cell number, using CyQuant reagent (Life Technologies). Metabolic assessments were conducted three separate times using primary PT cells derived from three mice. For calculations of metabolic parameters, results from each derivation were normalized as fold change versus DMSO and the replicates from all three derivations are shown.

### Assessment of Mitochondrial Content

To assess mitochondrial content, primary PT cells were cultured on coverslips and treated with DMSO, BT2, AAI, or AAI + BT2 for 24 h. Cells were washed with PBS and fixed with ice-cold 4% paraformaldehyde (PFA). After blocking with donkey serum, cells were stained with rabbit anti-translocase of the outer mitochondrial membrane (TOMM20) antibody (ab78547, 1:150 dilution; Abcam) followed by donkey anti-rabbit-568 (A10042; Life Technologies) and counterstained with Hoechst. Digital images were obtained with a Nikon Eclipse 90i microscope and DS-Qi2 camera. Mitochondrial area per cell was quantified as total area staining positive for TOMM20 divided by the number of nuclei using ImageJ software. For each group, three independent treatments were performed and the areas of 4–8 images per well were quantified for each treatment. The fold change versus DMSO for each of the treatment replicates is shown.

### Statistical Analysis

All data are expressed as means ± SE. Unpaired two-tailed Student’s *t* test was used to compare data between two groups, and ANOVA comparison with Tukey's/Dunn's post hoc test was done on multiple groups using GraphPad Prism 10. Differences were considered to be statistically significant when *P* < 0.05. Nonparametric statistical tests were performed using the Mann–Whitney *U* test to compare continuous data between two groups and Kruskal–Wallis test with Dunn’s post-test to compare continuous data between more than two groups. Details regarding statistical tests and *P* values can be found in the figure legends.

## RESULTS

### BT2 Treatment Attenuates Loss of Kidney Function in Nephrotoxic AKI

To induce AKI in mice, we first administered AAI injections with varying time intervals to determine the earliest time point at which the loss of kidney function and tubular injury could be observed (Supplemental Fig. S1). Two AAI injections at 3 days apart resulted in a significant reduction in kidney function, increased vimentin (*Vim*), and decreased E-cadherin (*Cdh1*) expression, and histological injury, compared to one injection with perfusion after 24 h or 48 h and controls (Supplemental Fig. S1). Immunofluorescent staining for Lotus lectin (PT brush borders) and cytokeratin-20 (KRT-20), a marker for PT injury, revealed significant reduction of Lotus lectin and increased KRT-20 after two injections of AAI (Supplemental Fig. S2, *A* and *B*). Moreover, mRNA expression of genes encoding BCAA catabolic enzymes showed significant downregulation following two AAI injections (Supplemental Fig. S2*C*). We therefore used two AAI injections to determine the protective effects of BT2.

To determine the effect of BT2 on kidney function in AKI, mice were treated daily with DMSO (vehicle) or BT2 in addition to the AAI (Fig. 1*A*). Control groups received daily treatments of either DMSO or BT2 without AAI. Inhibition of BCKDK by BT2 was confirmed by Western blotting for phospho-(p-)BCKDH-E1α in the kidney cortex. p-BCKDH-E1α was significantly reduced in uninjured mice treated with BT2, with a strong trend toward reduced p-BCKDH-E1α in injured kidneys (Supplemental Fig. S3, *A* and *B*). As expected, BT2 treatment did not result in changes in mRNA expression of enzymes in the BCAA catabolic pathway (Supplemental Fig. S3*C*). BT2 also did not significantly attenuate the decreased mRNA expression of the key FAO transcriptional regulator *Ppara* or two of its targets in the FAO pathway, *Cpt1a* and *Acaa2* (Supplemental Fig. S3*D*). Mice treated with BT2 in the control group exhibited a slightly lower body weight compared with those treated with DMSO, but this difference was not significant. In the AKI models, BT2 treatment led to slightly less body weight loss compared with the AAI + DMSO group, but this difference did not reach statistical significance (Fig. 1*B*). No significant differences in kidney weights were observed (Fig. 1*C*). Serum creatinine and urea nitrogen levels were significantly elevated in the AAI + DMSO group when compared with the control groups. However, these elevations were significantly mitigated with the administration of BT2 (Fig. 1, *D* and *E*). Histological evaluation revealed extensive tubular injuries, tubular cell death, loss of brush border, dilation of tubules, and protein casts within the AAI + DMSO group. However, these injuries were substantially mitigated with BT2 treatment in the AKI models (Fig. 1, *F* and *G*). Thus, loss of kidney function and histological injury in nephrotoxic AKI were partially prevented by BT2 administration.

### BT2 Treatment Attenuates the Loss of PT and Reduces Injury and Inflammation during AKI

**Figure 2.**
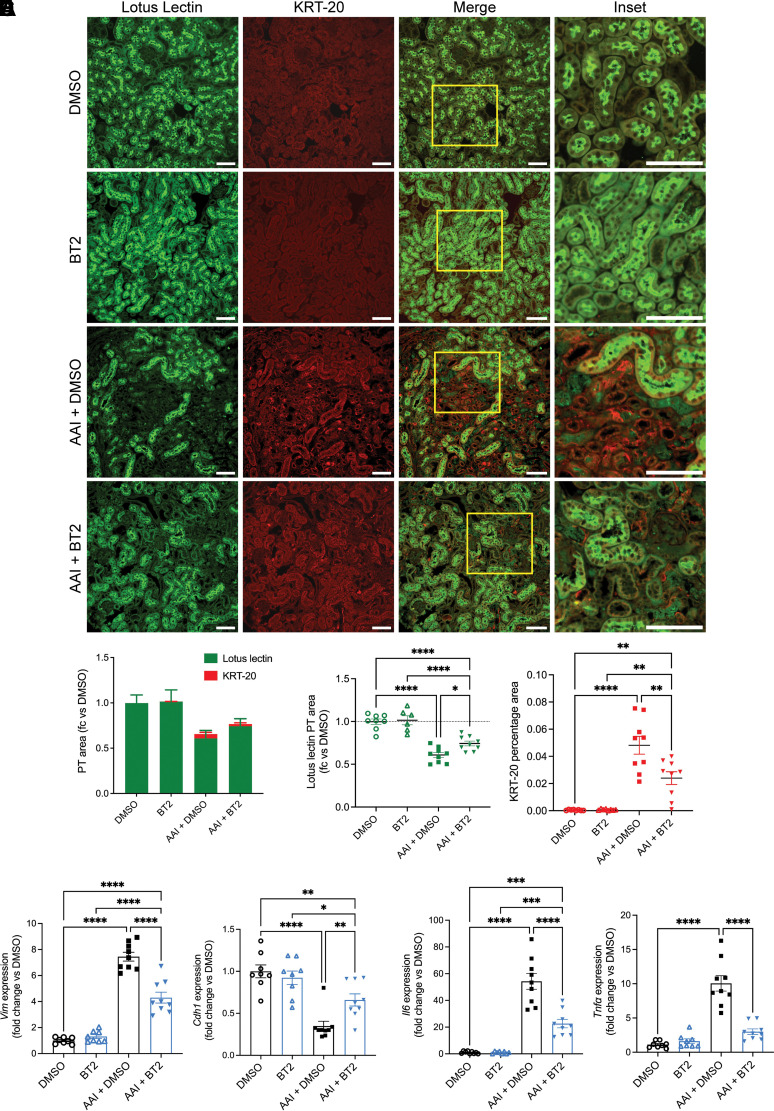
BT2 treatment reduces injury in nephrotoxic AKI. *A*: immunofluorescent staining for cytokeratin-20 (KRT-20) (injured PT; red) with counterstaining for Lotus lectin (healthy PT; green). Scale bars = 100 µm. *B* and *C*: quantification of area stained for Lotus lectin and KRT-20. *D* and *E*: kidney cortex mRNA expression levels of *Vim* (*D*) and *Cdh1* (*E*). *F*: kidney cortex mRNA expression of inflammatory markers *Il6* and *Tnfα*. AKI, acute kidney injury; PT, proximal tubule. *C*–*F*: *P < 0.05, **P < 0.01, ***P < 0.001, ****P < 0.0001, two-way ANOVA.

To investigate the effect of BT2 on PT cells, various tubular injury markers were assessed in the kidney cortex of AKI mice. Immunofluorescent staining of kidney sections demonstrated a significant loss of differentiated PT cells in the AAI + DMSO group, indicated by reduced Lotus lectin staining, in comparison with the control groups. However, this loss of PT cells was significantly alleviated in the AAI + BT2 group (Fig. 2, *A–C*). The area stained for KRT-20, indicative of injured PT cells, was significantly increased in the AAI + DMSO group but significantly reduced in the AAI + BT2 group (Fig. 2, *A–C*). Furthermore, we assessed the mRNA expression of injury markers *Vim* and *Cdh1*, across the different groups. As anticipated, we observed a substantial elevation in *Vim* in the AAI + DMSO group when compared with the control groups. This increase was significantly mitigated with BT2 treatment (Fig. 2*D*). *Cdh1* expression exhibited a significant decrease in the AAI + DMSO group, and this reduction was significantly counteracted by BT2 treatment in the AAI-injected mice (Fig. 2*E*). Inflammatory markers interleukin 6 (*Il6*) and tumor necrosis factor alpha (*Tnfa*) were also significantly increased in AAI-injected mice and significantly reduced by BT2 treatment (Fig. 2*F*). Thus, BT2 treatment significantly reduced PT cell loss and expression of injury and inflammatory markers in AKI models, indicating its potential protective effect against nephrotoxic PT injury.

### BT2 Reduces BCAA Accumulation and mTORC1 Signaling in Nephrotoxic AKI

**Figure 3.**
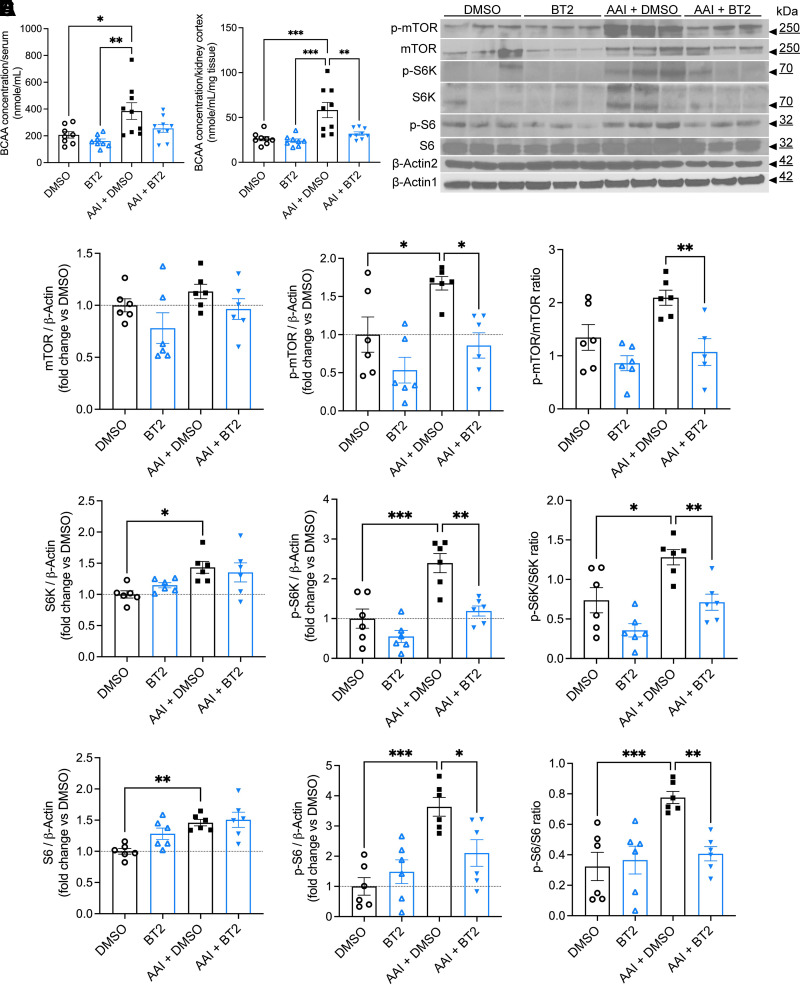
BCAA accumulation and mTORC1 signaling are reduced in BT2-treated mice. *A*: serum and kidney cortex BCAA concentrations. *B* and *C-E*: representative Western blots (*B*) and densitometry of the protein expression levels of mTOR and p-mTOR (*C*), S6K and p-S6K (*D*), S6 and p-S6 (*E*) in the kidney cortex lysates, all expression normalized versus β-Actin expression. β-Actin 1 is the control for total proteins, and β-Actin 2 is the control for phospho-proteins. BCAA, branched chain amino acid; mTORC1, mechanistic target of rapamycin complex 1. *A* and *C-E*: *P < 0.05, **P < 0.01, ***P < 0.001, one-way ANOVA.

Leucine is a potent stimulator of mTORC1 signaling which is known to be activated in AKI (11, 20–22). BCAAs, including leucine, have previously been shown to accumulate in other types of AKI (8), and BT2 treatment may alleviate this by increasing their catabolism. We therefore examined the accumulation of BCAA and mTORC1 signaling in the serum and kidney cortex of mice treated with AAI + BT2. Serum BCAA concentrations were significantly higher in AAI + DMSO-treated mice and showed a nonsignificant trend toward reduction in AAI + BT2-treated mice (Fig. 3*A*). There was a significant increase in kidney cortex BCAA concentrations in mice treated with AAI + DMSO, which was significantly attenuated in the group that received AAI + BT2 (Fig. 3*A*).

Examination of kidney cortex mTOR by Western blotting (Fig. 3*B*) showed that there was a significant increase in active phosphorylated mTOR, which was significantly reduced in mice treated with BT2 (Fig. 3*C*). Furthermore, the mTORC1 downstream pathway members S6 kinase (S6K) and S6 (S6) also had increased phosphorylation in the AAI + DMSO group and significant reduction of phosphorylation in the AAI + BT2 group (Fig. 3, *D* and *E*). Thus, accumulation of BCAA in the kidney cortex likely led to the activation of the mTORC1 signaling pathway, but BT2 treatment reduced BCAA accumulation and reduced mTORC1 signaling.

### BT2 Attenuates Defective Mitochondrial Bioenergetics and Cellular Injury in Vitro

**Figure 4.**
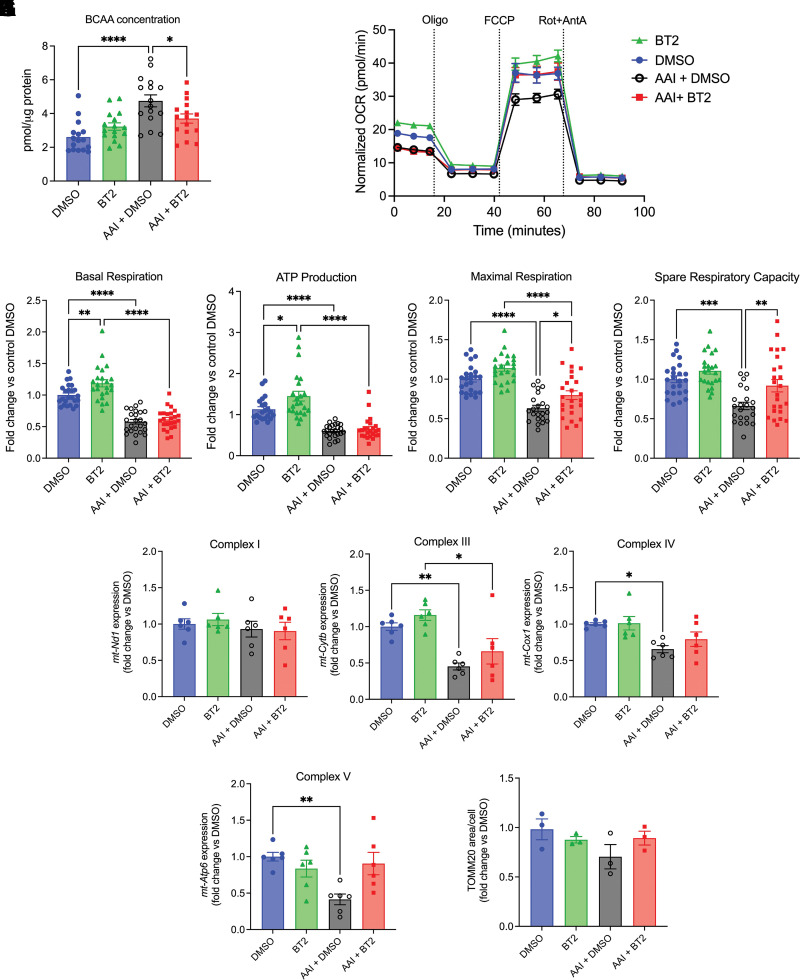
BT2 enhances BCAA catabolism and improves mitochondrial bioenergetics in primary PT cells. Primary PT cells were treated with DMSO or 25 µm AAI, with or without 25 µm BT2 for 24 h or 48 h. *A*: intracellular BCAA concentrations at 24 h (cells derived from three different mice). *B*: representative OCR at 24 h at baseline, and after addition of oligomycin (Oligo), FCCP, and combined rotenone plus antimycin A (Rot + AntA). *C*–*F*: quantification of basal respiration (*C*), ATP production (*D*), maximal respiration (*E*), and spare respiratory capacity (*F*), relative to DMSO (cells derived from three different mice). *G*–*J*: mRNA expression of *mt-Nd1* (complex I), *mt-Cytb* (complex III), *mt-Cox1* (complex IV), and *mt-Atp6* (complex V) at 24 h (cells derived from two different mice). *K*: quantification of area stained for TOMM20 per cell. AAI, aristolochic acid I; BCAA, branched chain amino acid; PT, proximal tubule; OCR, oxygen consumption rate. *A*, *C*–*K*: *P < 0.05, **P < 0.01, ***P < 0.001, ****P < 0.0001, one-way ANOVA.

We first evaluated the effects of BT2 to stimulate the catabolism of BCAA in primary PT cells. As expected, BCAAs accumulated in cells upon AAI treatment but this was significantly reduced by the addition of BT2, indicating increased catabolism and removal of BCAAs (Fig. 4*A*). BCAAs are used by the kidney to generate TCA cycle intermediates acetyl-CoA and succinyl-CoA. Therefore, we evaluated the effect of BT2 on mitochondrial respiration in primary PT cells using the Seahorse MitoStress test. Cells were treated with either DMSO, 25 μM BT2, 25 μM AAI, or a combination of AAI and BT2 for 24 h, and initial OCR was measured followed by the addition of oligomycin (ATP synthase inhibitor), FCCP (uncoupling agent) to measure maximal respiration, and rotenone/antimycin A (complex I and III inhibitors) (Fig. 4*B*). In uninjured cells, BT2 stimulated basal OCR and ATP production, likely indicating increased flux through the BCAA catabolic pathway to drive the TCA cycle (Fig. 4, *C–F*). AAI treatment resulted in significant reductions in basal respiration, ATP production, maximal respiration, and spare respiratory capacity compared to DMSO or BT2-treated cells (Fig. 4, *C*–*F*). BT2 did not rescue basal respiration or ATP production in AAI-treated cells but did significantly attenuate the reductions in maximal respiration and spare respiratory capacity compared to AAI-treated cells (Fig. 4, *C–F*). To determine whether these protective effects of BT2 were likely occurring through changes in general mitochondrial structure/function or BCAA pathway-specific effects, we undertook gene expression analysis of key mitochondrially encoded electron transport chain (ETC) subunits and measurement of mitochondrial content. Complex III (*mt-Cytb*), Complex IV (*mt-Cox1*), and Complex V (*mt-Atp6*) showed a significant reduction in expression with AAI treatment, but no significant increase with AAI plus BT2 treatment (Fig. 4, *G–J*). Mitochondrial content was assessed by immunofluorescence for translocase of the outer mitochondrial membrane 20 (TOMM20). There were no significant changes in total mitochondrial area per cell with either AAI or AAI plus BT2 treatment (Fig. 4*K*). These data suggest that BT2 partially protected mitochondrial bioenergetics in PT cells, likely through improved flux through the BCAA catabolic pathway.

**Figure 5.**
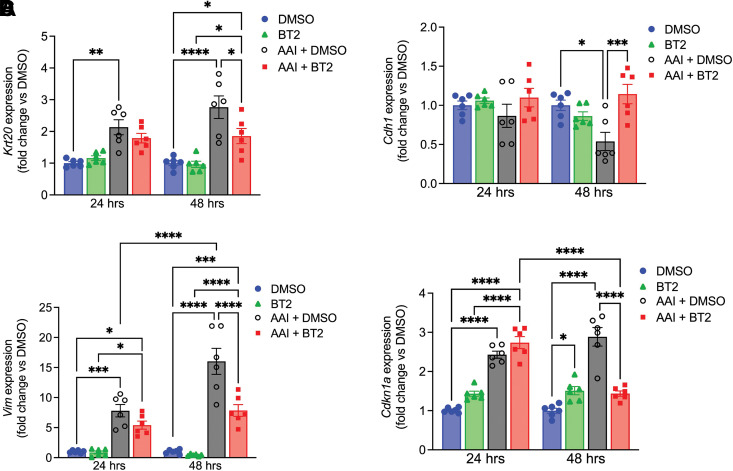
BT2 improves injury markers in primary proximal tubule (PT) cells. *A*–*D*: mRNA expression of *Krt20*, *Cdh1*, *Vim*, and *Cdkn1a* at 24 and 48 h (cells derived from two different mice). *P < 0.05, **P < 0.01, ***P < 0.001, ****P < 0.0001, two-way ANOVA.

We also assessed the effects of BT2 on injury in primary PT cells. We observed increased expression of *Krt20* in AAI-treated cells at 24 and 48 h post treatments, which was significantly reduced by BT2 treatment after 48 h (Fig. 5*A*). *Cdh1* expression significantly decreased and *Vim* expression increased in the AAI-treated group after 48 h indicating primary PT dedifferentiation but both showed significant improvement following BT2 treatment (Fig. 5, *B* and *C*). Furthermore, expression of the cell cycle inhibitor *Cdkn1a* (p21) was significantly upregulated in cells treated with AAI and reduced by BT2 treatment at 48 h (Fig. 5*D*). Thus, BT2 treatment improved mitochondrial bioenergetics leading to significantly attenuated PT injury, dedifferentiation, and cell cycle arrest.

## DISCUSSION

In this study, we demonstrate the protective role of pharmacological activation of BCAA catabolism by BT2 against nephrotoxic AKI in mice. BT2 has previously been shown to have effects on reducing hepatocellular carcinoma growth in mice in association with decreased mTORC1 signaling (12) and in alleviating heart failure in mice induced by transverse aortic constriction (17, 23, 24) and cardiac ischemia in association with decreased mTORC1 signaling (25–27). Our study is the first to show the beneficial effects of BT2 in preventing AKI, with significantly improved kidney function, reduced tubular injury, attenuated BCAA accumulation, and decreased mTORC1 signaling activation in the kidney. Notably, whereas BT2 induced a nonsignificant reduction of ∼33% in serum BCAA concentrations in AKI, we found a much larger and significant reduction of ∼45% in the kidney cortex with BT2, demonstrating a direct effect in reducing kidney cortex BCAA concentrations beyond the slight systemic effects of reduction in circulating BCAAs. Furthermore, BT2 improved mitochondrial bioenergetics and reduced injury in primary PT cells. Thus, the protective effect of BT2 appears to be mediated through two different cellular mechanisms: improved mitochondrial respiration and reduced mTORC1 signaling.

PT predominantly uses mitochondrial FAO to generate ATP via the TCA cycle and oxidative phosphorylation. However, AKI causes PT mitochondrial dysfunction regardless of the initial insult (28). In addition, it is well established that FAO is frequently downregulated in AKI through defective gene expression of key enzymes such as *Cpt1a* and *Acaa2* (5, 8, 19). Although BT2 treatment has been shown to upregulate the expression of genes encoding some enzymes in the FAO pathway in cardiac muscle (17) and liver (16), we did not detect significant increases in the expression of *Ppara*, *Cpt1a*, or *Acaa2* after BT2 treatment in the injured kidney cortex. However, further studies will be required to determine the expression of other enzymes, as well as flux through the FAO pathway after BT2 treatment. Uninjured kidneys also use BCAA to generate the TCA cycle intermediates succinyl-CoA and acetyl-CoA. Indeed, the kidney is one of the highest catabolizers of BCAA by organ weight (7). Furthermore, quantitative proteomics analysis of mouse tubular segments showed high levels of BCAA catabolic enzymes in the PT, indicating that a large proportion of kidney BCAA catabolism likely occurs in the PT (6). As such, BCAA catabolism would have the potential to help sustain the PT TCA cycle in the setting of AKI when FAO is downregulated. However, we and others have shown that BCAA catabolic enzymes are also transcriptionally downregulated in AKI (8–10), which results in the accumulation of BCAA in the kidney cortex (Fig. 3*A*). In normal primary PT cells, treatment with BT2 increased both basal mitochondrial respiration and ATP production after 24 h treatment, likely indicating increased BCAA catabolic flux. However, in injured PT cells, basal respiration and ATP production were not altered by BT2, whereas maximal respiration and spare respiratory capacity were significantly improved (Fig. 4). This was associated with decreased accumulation of BCAA in cells treated with AAI plus BT2 compared with AAI alone. Although basal respiration and ATP production did not change with BT2 treatment, the improvements in maximal and spare respiratory capacity with BT2 treatment indicate that BT2 was likely able to stimulate complete catabolism of BCAA to acetyl-CoA and succinyl-CoA. Consistent with this conclusion, mRNA expression of key mitochondrially encoded subunits of complexes III, IV, and V was not rescued by BT2, suggesting that the improvements in maximal respiration were likely due to improved availability of TCA cycle intermediates rather than the rescue of the ETC. Injured cells have lower requirements for mitochondrial respiration and ATP production due to disruption of energy-requiring processes such as solute transport. After 24 h, AAI plus BT2-treated cells had similar levels of injury markers to AAI-treated cells, which likely accounts for the lack of effect of BT2 on basal respiration and ATP production at this timepoint. By contrast, measurement of maximal respiration is uncoupled from the cellular ATP requirement and reflects the mitochondrial ability to rapidly use the available substrates. Furthermore, since injury markers were not different between AAI- and AAI + BT2-treated cells at 24 h, the improvements in maximal respiration and spare respiratory capacity were not due to BT2-treated cells having less injury. After 48 h, primary PT cell injury markers were significantly improved by BT2 treatment, mirroring the protective effect of BT2 in vivo, and suggesting that improvement of mitochondrial bioenergetics preceded the improvements in cellular injury. Thus, BT2 was able to partially restore mitochondrial bioenergetics in injured primary PT cells, and this was associated with subsequent improvement in injury markers.

We also showed that in vivo, BT2 restored mTORC1 signaling (p-mTOR, pS6K, pS6) to levels similar to those in uninjured mice (Fig. 3). Leucine is a potent activator of mTORC1, through at least three reported mechanisms. First, leucine can bind to sestrin 2, relieving its repression of GATOR2, which is then free to inhibit the mTORC1 inhibitor GATOR1 (29, 30). Second, leucine binds to leucyl-tRNA synthetase (LRS), which acts as a GTPase activating protein (GAP) for Rag GTPase, which activates mTORC1 (31, 32). Third, in the absence of leucine, secretion-associated Ras-related GTPase 1B (SAR1B) can also repress GATOR2, which is relieved in the presence of leucine (33). mTORC1 signaling is active in AKI of various etiologies including nephrotoxic, IRI, and septic (22, 34, 35). The relationship between leucine accumulation and mTORC1 activity has not directly been studied in AKI, but our data showing that BT2 reduces both BCAA concentrations and mTORC1 activity strongly suggests that excess leucine contributes to mTORC1 hyperactivity after AAI. Further studies will be needed to determine which leucine-sensing mechanism(s) may account for mTORC1 hyperactivity in AKI. The presence of mTORC1 as part of TOR-autophagy spatial coupling compartments (TASCCs) is associated with the secretion of profibrotic factors, cell cycle arrest at the G2/M checkpoint, and exacerbated injury in nephrotoxic (AAI) and IRI-induced AKI and fibrosis (20). However, there are conflicting data concerning the effects of mTOR inhibition by rapamycin in AKI, stemming from different timings, doses, and injury models used in published studies (20, 36–39). Thus, inhibition of mTOR early in injury appears to worsen AKI, whereas later inhibition is associated with improved repair. This suggests that a delicate balance of the level of mTORC1 activity and timing is crucial for outcomes after AKI and that subtle alterations in mTORC1 activity rather than strong inhibition may constitute an improved therapeutic strategy. Consistent with this hypothesis, complete loss of PT mTORC1 activity caused a renal Fanconi-like syndrome (40) and exacerbated AKI (21), whereas partial inhibition improved kidney function and fibrosis in diabetic Akita mice (41). Intriguingly, a recent study of mice with aberrant expression of PT amino acid transporters due to knockout of collectrin also found attenuation of mTORC1 signaling associated with improved pathology in a diabetic kidney disease (DKD) model (42). Of note, mTORC1 signaling influences many downstream pathways in addition to S6K and S6 and further studies will be needed to determine whether any of them are also altered by BT2 treatment.

Here, we studied the effects of BT2 in preventing nephrotoxic injury using AAI. It will be important to determine whether BT2 may have beneficial effects after the onset of AKI, as well as in other types of AKI. For example, BCAA enzymes were also found to be transcriptionally downregulated in IRI, suggesting that BT2 could have beneficial effects (8). A recent study showed only mild attenuations in mRNA levels of inflammatory and ferroptotic markers and no differences in fibrotic gene expression in mice injured with repeated low-dose cisplatin and treated with twice weekly BT2 administration (43). However disturbances in BCAA enzymes expression and accumulation of BCAA were shown in unilateral ureteral obstruction (UUO) mice (8), so further studies will be needed to define the effects of BT2 in other fibrotic injury models, as well as DKD, and other kidney diseases with hyperactivation of mTORC1, such as autosomal dominant polycystic kidney disease (ADPKD) (44).

In conclusion, our study highlights the potential of activating BCAA catabolism as a therapeutic strategy for AKI. By enhancing BCAA catabolism, BT2 reduced toxic BCAA accumulation, alleviated mTORC1 signaling, and improved mitochondrial function, collectively contributing to the preservation of kidney function and reduction of PT cellular injury. Thus, pharmacological activation of BCAA catabolism with BT2 offers a promising approach to attenuate the adverse effects of AKI. Further studies are needed to explore the clinical applicability of this strategy, for example, in treating AKI, and to better understand the underlying mechanisms through which BCAA catabolism influences kidney injury and repair processes.

## DATA AVAILABILITY

Data will be made available upon reasonable request.

## SUPPLEMENTAL MATERIAL

10.6084/m9.figshare.27775011.v1 Supplemental Tables S1 and S2: https://doi.org/10.6084/m9.figshare.27775011.v1.

10.6084/m9.figshare.27774741.v1 Supplemental Fig. S1: https://doi.org/10.6084/m9.figshare.27774741.v1.

10.6084/m9.figshare.27774894.v1 Supplemental Fig. S2: https://doi.org/10.6084/m9.figshare.27774894.v1.

10.6084/m9.figshare.27774942.v1 Supplemental Fig. S3: https://doi.org/10.6084/m9.figshare.27774942.v1.

## GRANTS

This work was supported by an NYC Train KUHR U2C-TL1 Postdoctoral Training Fellowship (to S.D.), an American Society of Nephrology KidneyCure Joseph V. Bonventre Research Scholar Award (to S.E.P.), American Heart Association Career Development Award (to S.E.P.), NIH-NIDDK Grants [No. DK133238 (to S.E.P.), No. DK112984 (to S.K.M.), and No. DK121846 (to S.K.M.)], Veterans Affairs [I01BX003698 (to S.K.M.) and IS1BX004815 (to S.K.M.)], and Dialysis Clinic Inc. (to S.E.P. and S.K.M.).

## DISCLOSURES

No conflicts of interest, financial or otherwise, are declared by the authors.

## AUTHOR CONTRIBUTIONS

S.D., S.K.M., and S.E.P. conceived and designed research; S.D. and S.E.P. performed experiments; S.D., M.P.R., and S.E.P. analyzed data; S.D., S.K.M., and S.E.P. interpreted results of experiments; S.D. and S.E.P. prepared figures; S.D. drafted manuscript; S.D., S.K.M., and S.E.P. edited and revised manuscript; S.D., S.K.M., and S.E.P. approved final version of manuscript.

## References

[1] Chawla LS, Bellomo R, Bihorac A, Goldstein SL, Siew ED, Bagshaw SM, Bittleman D, Cruz D, Endre Z, Fitzgerald RL, Forni L, Kane-Gill SL, Hoste E, Koyner J, Liu KD, Macedo E, Mehta R, Murray P, Nadim M, Ostermann M, Palevsky PM, Pannu N, Rosner M, Wald R, Zarbock A, Ronco C, Kellum JA; Acute Disease Quality Initiative Workgroup 16. Acute kidney disease and renal recovery: consensus report of the Acute Disease Quality Initiative (ADQI) 16 Workgroup. Nat Rev Nephrol 13: 241–257, 2017. doi:10.1038/nrneph.2017.2. 28239173

[2] Chang-Panesso M, Humphreys BD. Cellular plasticity in kidney injury and repair. Nat Rev Nephrol 13: 39–46, 2017. doi:10.1038/nrneph.2016.169. 27890924

[3] Yu SM, Bonventre JV. Acute kidney injury and maladaptive tubular repair leading to renal fibrosis. Curr Opin Nephrol Hypertens 29: 310–318, 2020. doi:10.1097/MNH.0000000000000605. 32205583 PMC7363449

[4] Hall AM, de Seigneux S. Metabolic mechanisms of acute proximal tubular injury. Pflugers Arch 474: 813–827, 2022. doi:10.1007/s00424-022-02701-y. 35567641 PMC9338906

[5] Kang HM, Ahn SH, Choi P, Ko YA, Han SH, Chinga F, Park AS, Tao J, Sharma K, Pullman J, Bottinger EP, Goldberg IJ, Susztak K. Defective fatty acid oxidation in renal tubular epithelial cells has a key role in kidney fibrosis development. Nat Med 21: 37–46, 2015. doi:10.1038/nm.3762. 25419705 PMC4444078

[6] Lyu Z, Mao Z, Li Q, Xia Y, Liu Y, He Q, Wang Y, Zhao H, Lu Z, Zhou Q. PPARγ maintains the metabolic heterogeneity and homeostasis of renal tubules. EBioMedicine 38: 178–190, 2018. doi:10.1016/j.ebiom.2018.10.072. 30420298 PMC6306377

[7] Neinast MD, Jang C, Hui S, Murashige DS, Chu Q, Morscher RJ, Li X, Zhan L, White E, Anthony TG, Rabinowitz JD, Arany Z. Quantitative analysis of the whole-body metabolic fate of branched-chain amino acids. Cell Metab 29: 417–429.e4, 2019. doi:10.1016/j.cmet.2018.10.013. 30449684 PMC6365191

[8] Li H, Dixon EE, Wu H, Humphreys BD. Comprehensive single-cell transcriptional profiling defines shared and unique epithelial injury responses during kidney fibrosis. Cell Metab 34: 1977–1998.e9, 2022. doi:10.1016/j.cmet.2022.09.026. 36265491 PMC9742301

[9] Piret SE, Guo Y, Attallah AA, Horne SJ, Zollman A, Owusu D, Henein J, Sidorenko VS, Revelo MP, Hato T, Ma'ayan A, He JC, Mallipattu SK. Krüppel-like factor 6-mediated loss of BCAA catabolism contributes to kidney injury in mice and humans. Proc Natl Acad Sci USA 118: e2024414118, 2021. doi:10.1073/pnas.2024414118. 34074766 PMC8201852

[10] Standage SW, Xu S, Brown L, Ma Q, Koterba A, Lahni P, Devarajan P, Kennedy MA. NMR-based serum and urine metabolomic profile reveals suppression of mitochondrial pathways in experimental sepsis-associated acute kidney injury. Am J Physiol Renal Physiol 320: F984–F1000, 2021. doi:10.1152/ajprenal.00582.2020. 33843271 PMC8424559

[11] Neinast M, Murashige D, Arany Z. Branched chain amino acids. Annu Rev Physiol 81: 139–164, 2019. doi:10.1146/annurev-physiol-020518-114455. 30485760 PMC6536377

[12] Ericksen RE, Lim SL, McDonnell E, Shuen WH, Vadiveloo M, White PJ, Ding Z, Kwok R, Lee P, Radda GK, Toh HC, Hirschey MD, Han W. Loss of BCAA catabolism during carcinogenesis enhances mTORC1 activity and promotes tumor development and progression. Cell Metab 29: 1151–1165.e6, 2019. doi:10.1016/j.cmet.2018.12.020. 30661928 PMC6506390

[13] Tso S-C, Gui W-J, Wu C-Y, Chuang JL, Qi X, Skvora KJ, Dork K, Wallace AL, Morlock LK, Lee BH, Hutson SM, Strom SC, Williams NS, Tambar UK, Wynn RM, Chuang DT. Benzothiophene carboxylate derivatives as novel allosteric inhibitors of branched-chain α-ketoacid dehydrogenase kinase. J Biol Chem 289: 20583–20593, 2014. doi:10.1074/jbc.M114.569251. 24895126 PMC4110271

[14] White PJ, McGarrah RW, Grimsrud PA, Tso SC, Yang WH, Haldeman JM, Grenier-Larouche T, An J, Lapworth AL, Astapova I, Hannou SA, George T, Arlotto M, Olson LB, Lai M, Zhang GF, Ilkayeva O, Herman MA, Wynn RM, Chuang DT, Newgard CB. The BCKDH kinase and phosphatase integrate BCAA and lipid metabolism via regulation of ATP-citrate lyase. Cell Metab 27: 1281–1293.e7, 2018. doi:10.1016/j.cmet.2018.04.015. 29779826 PMC5990471

[15] Yang Y, Wang S, Sheng C, Tan J, Chen J, Li T, Ma X, Sun H, Wang X, Zhou L. Branched-chain amino acid catabolic defect promotes α-cell proliferation via activating mTOR signaling. Mol Cell Endocrinol 582: 112143, 2024. doi:10.1016/j.mce.2023.112143. 38158148

[16] Bollinger E, Peloquin M, Libera J, Albuquerque B, Pashos E, Shipstone A, Hadjipanayis A, Sun Z, Xing G, Clasquin M, Stansfield JC, Tierney B, Gernhardt S, Siddall CP, Greizer T, Geoly FJ, Vargas SR, Gao LC, Williams G, Marshall M, Rosado A, Steppan C, Filipski KJ, Zhang BB, Miller RA, Roth Flach RJ. BDK inhibition acts as a catabolic switch to mimic fasting and improve metabolism in mice. Mol Metab 66: 101611, 2022. doi:10.1016/j.molmet.2022.101611. 36220546 PMC9589198

[17] Chen M, Gao C, Yu J, Ren S, Wang M, Wynn RM, Chuang DT, Wang Y, Sun H. Therapeutic effect of targeting branched-chain amino acid catabolic flux in pressure-overload induced heart failure. J Am Heart Assoc 8: e011625, 2019. doi:10.1161/JAHA.118.011625. 31433721 PMC6585363

[18] Anger EE, Yu F, Li J. Aristolochic acid-induced nephrotoxicity: molecular mechanisms and potential protective approaches. Int J Mol Sci 21: 1157, 2020. doi:10.3390/ijms21031157. 32050524 PMC7043226

[19] Piret SE, Attallah AA, Gu X, Guo Y, Gujarati NA, Henein J, Zollman A, Hato T, Ma'ayan A, Revelo MP, Dickman KG, Chen C-H, Shun CT, Rosenquist TA, He JC, Mallipattu SK. Loss of proximal tubular transcription factor Krüppel-like factor 15 exacerbates kidney injury through loss of fatty acid oxidation. Kidney Int 100: 1250–1267, 2021. doi:10.1016/j.kint.2021.08.031. 34634362 PMC8608748

[20] Canaud G, Brooks CR, Kishi S, Taguchi K, Nishimura K, Magassa S, Scott A, Hsiao LL, Ichimura T, Terzi F, Yang L, Bonventre JV. Cyclin G1 and TASCC regulate kidney epithelial cell G(2)-M arrest and fibrotic maladaptive repair. Sci Transl Med 11: eaav4754, 2019. doi:10.1126/scitranslmed.aav4754. 30674655 PMC6527117

[21] Grahammer F, Haenisch N, Steinhardt F, Sandner L, Roerden M, Arnold F, Cordts T, Wanner N, Reichardt W, Kerjaschki D, Ruegg MA, Hall MN, Moulin P, Busch H, Boerries M, Walz G, Artunc F, Huber TB. mTORC1 maintains renal tubular homeostasis and is essential in response to ischemic stress. Proc Natl Acad Sci USA 111: E2817–E2826, 2014. doi:10.1073/pnas.1402352111. 24958889 PMC4103333

[22] Wang Y, Liu Z, Shu S, Cai J, Tang C, Dong Z. AMPK/mTOR signaling in autophagy regulation during cisplatin-induced acute kidney injury. Front Physiol 11: 619730, 2020. doi:10.3389/fphys.2020.619730. 33391038 PMC7773913

[23] Sun H, Olson KC, Gao C, Prosdocimo DA, Zhou M, Wang Z, Jeyaraj D, Youn JY, Ren S, Liu Y, Rau CD, Shah S, Ilkayeva O, Gui WJ, William NS, Wynn RM, Newgard CB, Cai H, Xiao X, Chuang DT, Schulze PC, Lynch C, Jain MK, Wang Y. Catabolic defect of branched-chain amino acids promotes heart failure. Circulation 133: 2038–2049, 2016. doi:10.1161/CIRCULATIONAHA.115.020226. 27059949 PMC4879058

[24] Uddin GM, Zhang L, Shah S, Fukushima A, Wagg CS, Gopal K, Al Batran R, Pherwani S, Ho KL, Boisvenue J, Karwi QG, Altamimi T, Wishart DS, Dyck JRB, Ussher JR, Oudit GY, Lopaschuk GD. Impaired branched chain amino acid oxidation contributes to cardiac insulin resistance in heart failure. Cardiovasc Diabetol 18: 86, 2019. doi:10.1186/s12933-019-0892-3. 31277657 PMC6610921

[25] Li T, Zhang Z, Kolwicz SC Jr, Abell L, Roe ND, Kim M, Zhou B, Cao Y, Ritterhoff J, Gu H, Raftery D, Sun H, Tian R. Defective branched-chain amino acid catabolism disrupts glucose metabolism and sensitizes the heart to ischemia-reperfusion injury. Cell Metab 25: 374–385, 2017. doi:10.1016/j.cmet.2016.11.005. 28178567 PMC5301464

[26] Murashige D, Jung JW, Neinast MD, Levin MG, Chu Q, Lambert JP, Garbincius JF, Kim B, Hoshino A, Marti-Pamies I, McDaid KS, Shewale SV, Flam E, Yang S, Roberts E, Li L, Morley MP, Bedi KC Jr, Hyman MC, Frankel DS, Margulies KB, Assoian RK, Elrod JW, Jang C, Rabinowitz JD, Arany Z. Extra-cardiac BCAA catabolism lowers blood pressure and protects from heart failure. Cell Metab 34: 1749–1764.e7, 2022. doi:10.1016/j.cmet.2022.09.008. 36223763 PMC9633425

[27] Wang W, Zhang F, Xia Y, Zhao S, Yan W, Wang H, Lee Y, Li C, Zhang L, Lian K, Gao E, Cheng H, Tao L. Defective branched chain amino acid catabolism contributes to cardiac dysfunction and remodeling following myocardial infarction. Am J Physiol Heart Circ Physiol 311: H1160–H1169, 2016. doi:10.1152/ajpheart.00114.2016. 27542406

[28] Jiang M, Bai M, Lei J, Xie Y, Xu S, Jia Z, Zhang A. Mitochondrial dysfunction and the AKI-to-CKD transition. Am J Physiol Renal Physiol 319: F1105–F1116, 2020. doi:10.1152/ajprenal.00285.2020. 33073587

[29] Valenstein ML, Rogala KB, Lalgudi PV, Brignole EJ, Gu X, Saxton RA, Chantranupong L, Kolibius J, Quast JP, Sabatini DM. Structure of the nutrient-sensing hub GATOR2. Nature 607: 610–616, 2022. doi:10.1038/s41586-022-04939-z. 35831510 PMC9464592

[30] Wolfson RL, Chantranupong L, Saxton RA, Shen K, Scaria SM, Cantor JR, Sabatini DM. Sestrin2 is a leucine sensor for the mTORC1 pathway. Science 351: 43–48, 2016. doi:10.1126/science.aab2674. 26449471 PMC4698017

[31] Han JM, Jeong SJ, Park MC, Kim G, Kwon NH, Kim HK, Ha SH, Ryu SH, Kim S. Leucyl-tRNA synthetase is an intracellular leucine sensor for the mTORC1-signaling pathway. Cell 149: 410–424, 2012. doi:10.1016/j.cell.2012.02.044. 22424946

[32] Kim S, Yoon I, Son J, Park J, Kim K, Lee JH, Park SY, Kang BS, Han JM, Hwang KY, Kim S. Leucine-sensing mechanism of leucyl-tRNA synthetase 1 for mTORC1 activation. Cell Rep 35: 109031, 2021. doi:10.1016/j.celrep.2021.109031. 33910001

[33] Chen J, Ou Y, Luo R, Wang J, Wang D, Guan J, Li Y, Xia P, Chen PR, Liu Y. SAR1B senses leucine levels to regulate mTORC1 signalling. Nature 596: 281–284, 2021. doi:10.1038/s41586-021-03768-w. 34290409

[34] He FF, Wang YM, Chen YY, Huang W, Li ZQ, Zhang C. Sepsis-induced AKI: From pathogenesis to therapeutic approaches. Front Pharmacol 13: 981578, 2022. doi:10.3389/fphar.2022.981578. 36188562 PMC9522319

[35] Zhao Y, Feng X, Li B, Sha J, Wang C, Yang T, Cui H, Fan H. Dexmedetomidine protects against lipopolysaccharide-induced acute kidney injury by enhancing autophagy through inhibition of the PI3K/AKT/mTOR pathway. Front Pharmacol 11: 128, 2020. doi:10.3389/fphar.2020.00128. 32158395 PMC7052304

[36] Jiang M, Wei Q, Dong G, Komatsu M, Su Y, Dong Z. Autophagy in proximal tubules protects against acute kidney injury. Kidney Int 82: 1271–1283, 2012. doi:10.1038/ki.2012.261. 22854643 PMC3491167

[37] Lieberthal W, Fuhro R, Andry CC, Rennke H, Abernathy VE, Koh JS, Valeri R, Levine JS. Rapamycin impairs recovery from acute renal failure: role of cell-cycle arrest and apoptosis of tubular cells. Am J Physiol Renal Physiol 281: F693–F706, 2001. doi:10.1152/ajprenal.2001.281.4.F693. 11553517

[38] Lui SL, Chan KW, Tsang R, Yung S, Lai KN, Chan TM. Effect of rapamycin on renal ischemia-reperfusion injury in mice. Transpl Int 19: 834–839, 2006. doi:10.1111/j.1432-2277.2006.00361.x. 16961776

[39] Sunahara S, Watanabe E, Hatano M, Swanson PE, Oami T, Fujimura L, Teratake Y, Shimazui T, Lee C, Oda S. Influence of autophagy on acute kidney injury in a murine cecal ligation and puncture sepsis model. Sci Rep 8: 1050, 2018. doi:10.1038/s41598-018-19350-w. 29348412 PMC5773584

[40] Grahammer F, Ramakrishnan SK, Rinschen MM, Larionov AA, Syed M, Khatib H, Roerden M, Sass JO, Helmstaedter M, Osenberg D, Kühne L, Kretz O, Wanner N, Jouret F, Benzing T, Artunc F, Huber TB, Theilig F. mTOR regulates endocytosis and nutrient transport in proximal tubular cells. J Am Soc Nephrol 28: 230–241, 2017. doi:10.1681/ASN.2015111224. 27297946 PMC5198276

[41] Kogot-Levin A, Hinden L, Riahi Y, Israeli T, Tirosh B, Cerasi E, Mizrachi EB, Tam J, Mosenzon O, Leibowitz G. Proximal tubule mTORC1 is a central player in the pathophysiology of diabetic nephropathy and its correction by SGLT2 inhibitors. Cell Rep 32: 107954, 2020. doi:10.1016/j.celrep.2020.107954. 32726619 PMC7397516

[42] Kano Y, Yamaguchi S, Mise K, Kawakita C, Onishi Y, Kurooka N, Sugawara R, Albuayjan HHH, Nakatsuka A, Eguchi J, Wada J. Inhibition of amino acids influx into proximal tubular cells improves lysosome function in diabetes. Kidney360 5: 182–194, 2024. doi:10.34067/KID.0000000000000333. 38062578 PMC10914197

[43] Sone H, Lee TJ, Lee BR, Heo D, Oh S, Kwon SH. MicroRNA-mediated attenuation of branched-chain amino acid catabolism promotes ferroptosis in chronic kidney disease. Nat Commun 14: 7814, 2023. doi:10.1038/s41467-023-43529-z. 38016961 PMC10684653

[44] Yamamoto J, Nishio S, Hattanda F, Nakazawa D, Kimura T, Sata M, Makita M, Ishikawa Y, Atsumi T. Branched-chain amino acids enhance cyst development in autosomal dominant polycystic kidney disease. Kidney Int 92: 377–387, 2017. doi:10.1016/j.kint.2017.01.021. 28341273

